# Persistent Oxidation of Mitochondrial and Transmembrane Proteins in Rat Cerebrum and Heart Regardless of Age or Nutrition

**DOI:** 10.3390/ijms262211155

**Published:** 2025-11-18

**Authors:** Wangya Yang, Shipan Fan, Carina Ramallo-Guevara, Manuela Kratochwil, Sandra Thilmany, Michiru D. Sugawa, Norbert A. Dencher, Ansgar Poetsch

**Affiliations:** 1 School of Basic Medical Sciences, Institute of Biomedical Innovation, Jiangxi Medical College, Nanchang University, Nanchang 330031, China; 2 Plant Biochemistry, Faculty of Biology & Biotechnology, Ruhr University Bochum, D-44801 Bochum, Germany; 3 Physical Biochemistry, Department of Chemistry, Technical University of Darmstadt, D-64287 Darmstadt, Germany; 4 Clinical Neurobiology, Department of Psychiatry, Campus Benjamin Franklin (CBF), Charite’ Universitätsmedizin Berlin, D-12203 Berlin, Germany

**Keywords:** reactive oxygen species, oxidative post-translational modifications, mitochondria, calorie restriction, aging, rat cerebrum, rat heart

## Abstract

Reactive oxygen species (ROS), inevitable by-products of aerobic metabolism, act both as regulators of signaling pathways and as mediators of oxidative stress and aging-related damage. Protein oxidative post-translational modifications (Ox-PTMs) are recognized hallmarks of aging and metabolic decline, yet the persistence of protein oxidation under different physiological conditions, such as age and diet, remains unclear. Here, we applied proteomics to mitochondrial and membrane-enriched fractions of male Fischer 344 rat cerebrum and heart, comparing Ox-PTMs across young and aged animals subjected to ad libitum nutrition (AL) or calorie restriction (CR). We identified 139 mitochondrial and membrane-associated proteins consistently exhibiting high levels of oxidation, including tricarboxylic acid (TCA) cycle enzymes, respiratory chain subunits, ATP synthase components, cytoskeletal proteins, and synaptic vesicle regulators. Functional enrichment and network analyses revealed that oxidized proteins clustered in modules related to mitochondrial energy metabolism, membrane transport, and excitation–contraction coupling. Notably, many proteins remained persistently oxidized, predominantly as mono-oxidation, without significant changes during aging or CR. Moreover, the enzymatic activity of mitochondrial complexes was not only preserved but significantly enhanced in specific contexts, and the structural integrity of the respiratory chain was maintained. These findings indicate a dual strategy for coping with oxidative stress: CR reduces ROS production to limit oxidative burden, while protein and network robustness enable functional adaptation to persistent oxidation, collectively shaping mitochondrial function and cellular homeostasis under differing physiological conditions.

## 1. Introduction

Reactive oxygen species (ROS) are reactive molecules generated as by-products of aerobic metabolism, primarily in mitochondria. They include radical species such as superoxide anions (O_2_•^−^) and hydroxyl radicals (•OH), and non-radical molecules such as hydrogen peroxide (H_2_O_2_). At physiological levels, ROS act as signaling mediators that regulate oxidative post-translational modifications (Ox-PTMs) on redox-sensitive proteins, modulating structure, localization, and function [1,2,3]. However, excessive ROS accumulation leads to oxidative stress (OS), damaging proteins, lipids, and nucleic acids, thereby contributing to neurodegenerative and cardiovascular diseases [4,5,6]. Ox-PTMs —including methionine oxidation, carbonylation, and tryptophan-to-kynurenine conversion—are widely recognized as molecular hallmarks of aging and disease [7,8,9,10].

Aging is an irreversible physiological process accompanied by functional decline and an increased risk of chronic disorders [11,12]. The mitochondrial free radical theory of aging (MFRTA) proposes that ROS derived from mitochondrial respiration cause cumulative macromolecular damage that drives aging [13]. Supporting evidence includes mitochondrial dysfunction, reduced respiratory capacity, and elevated oxidative damage observed in aged tissues [14,15]. Nevertheless, recent studies have refined this model, showing that ROS are not solely detrimental; moderate ROS signaling can enhance stress resistance and longevity through adaptive responses [16,17]. For instance, interventions that modulate energy metabolism, such as calorie restriction (CR), can extend lifespan partly by optimizing mitochondrial efficiency and redox balance [18]. These findings have shifted the focus from ROS toxicity to redox signaling and homeostasis as a determinant of aging.

Mitochondria serve as both the main source and target of ROS, making them central to cellular redox regulation [19,20]. With aging, increased mitochondrial ROS production, impaired electron transport efficiency, and weakened antioxidant capacity lead to site-specific oxidative modifications in mitochondrial and membrane proteins [21]. Proteomic studies have identified recurrently oxidized enzymes within the tricarboxylic acid (TCA) cycle (e.g., aconitase and malate dehydrogenase) and respiratory complexes (e.g., NDUF subunits of Complex I, COX1/2, and ATP synthase) [22,23,24]. Oxidative modifications on membrane transporters such as voltage-dependent anion channels (VDACs) and adenine nucleotide translocators (ANTs) alter metabolite flux and mitochondrial permeability, contributing to age-related dysfunction [25,26]. Redox-sensitive regulatory proteins, including mitofusin-2 (MFN2) and optic atrophy protein 1 (OPA1), also undergo thiol oxidation that impairs mitochondrial fusion and proteostasis [27]. Yet, it remains unclear whether mitochondrial protein oxidation occurs exclusively as an age-related process or partly reflects adaptive, age-independent redox responses, suggesting possible functional tolerance to ROS. Moreover, recent proteomic evidence indicates that not all mitochondrial proteins are equally prone to oxidation, suggesting selective vulnerability depending on protein localization, redox reactivity, and structural role [28,29]. Collectively, these observations highlight that certain mitochondrial and transmembrane proteins are more susceptible to oxidative modification than others, and that oxidation is not directly linked to functional decline during aging.

CR, defined as reduced energy intake without malnutrition, remains the most effective non-genetic intervention to delay aging and extend lifespan across species [30,31,32]. In rodents, 20–50% CR prolongs median and maximal lifespan and mitigates multiple age-related diseases [33]. CR improves mitochondrial efficiency, lowers ROS emission, and enhances antioxidant defenses, thereby maintaining proteostasis [34]. Proteomic analyses have shown that CR reduces oxidative damage to mitochondrial proteins such as ATP synthase, aconitase, and ETC subunits, preserving enzyme activity and Fe–S cluster integrity [22,35,36]. Moreover, CR triggers mild, transient ROS elevation that activates adaptive signaling, including Nrf2-mediated antioxidant responses [37]. These dual effects—ROS suppression and controlled ROS signaling—collectively underpin the beneficial impact of CR on mitochondrial function and longevity [38]. Nevertheless, despite global reductions in oxidation, certain proteins remain persistently oxidized under CR, suggesting intrinsic tolerance or functional significance of these modifications [39]. Obesity and metabolic overload represent the opposite end of the energy balance spectrum compared with CR. Chronic nutrient excess promotes mitochondrial substrate overload and disrupts redox homeostasis, resulting in sustained ROS overproduction and oxidative damage to lipids, proteins, and nucleic acids [40]. Elevated ROS levels in obesity have been implicated in impaired insulin signaling, endoplasmic reticulum stress, and mitochondrial dysfunction, which collectively accelerate cellular aging and metabolic decline [41]. These alterations compromise mitochondrial integrity and energy efficiency, thereby reinforcing a vicious cycle between oxidative stress and metabolic dysregulation [42].

Although ROS levels and oxidative damage can be modulated by age and diet, there may exist certain proteins that commonly exhibit persistently high oxidation. However, the biological significance of these specific oxidative modifications remains poorly understood, and their prevalence and functional impact under different metabolic conditions have yet to be systematically explored. To address this gap, we conducted a comparative redox–proteomic analysis of mitochondrial and transmembrane proteins in rat cerebrum and heart tissues from young and old animals fed ad libitum (AL) or under short or long-term CR. By integrating quantitative mass spectrometry with bioinformatics and biochemical validation, we identified 139 proteins consistently exhibiting high levels of mono-oxidation regardless of age or CR. Functional and network analyses revealed enrichment in modules associated with mitochondrial energy metabolism, membrane transport, and excitation–contraction coupling, indicating structural, functional, and network-level resilience to oxidative pressure. Our findings provide new insight into the selective vulnerability and intrinsic redox tolerance of mitochondrial and membrane proteins, revealing a previously underappreciated dimension of oxidative proteostasis in aging and metabolic adaptation.

## 2. Results

Male Fischer rats of F344 were subjected to short-term (Y) or long-term (O) ad libitum (AL) or calorie restriction (CR), and tissue samples from the heart and cerebrum were collected for the isolation of crude mitochondrial components. Consistent with previous studies, under CR animals exhibited lower body weights compared to age-matched AL-fed controls (Table S1), confirming the physiological impact of dietary intervention. Samples were profiled for proteomics and were grouped according to Ox-PTMs (mono-oxidation, oxidation (M), pyrrolidone, carbonylation, kynurenine, and 2-amino-3-ketobutyric acid) using LC-MS/MS. MaxQuant mass shift search was used for PTM identification and spectral counts for quantification. To gain a broader perspective on Ox-PTMs landscapes between two tissues, we conducted a principal component analysis (PCA) based on the spectral counts of three representative and readily quantifiable Ox-PTMs: mono-oxidation, carbonylation, and oxidation (M). The data from two tissues were integrated into this analysis, while the detection and comparability of three other Ox-PTMs were found to be less reliable. The integrated PCA plot revealed a clear separation between heart and cerebrum samples along the principal component that captured the majority of the variance (Figures S7–S9), demonstrating that tissue origin is the dominant factor shaping the profile of these oxidative modifications. Within the distinct tissue clusters, samples from different experimental groups (YAL, YCR, OAL, and OCR) were intermingled, indicating that the global patterns of these specific Ox-PTMs were not substantially reconfigured by aging or CR. The UniProt database was used for screening mitochondrial proteins or other transmembrane domain proteins. Proteins with an oxidation rate of ≥0.8 in each group were retained and were subjected to subsequent bioinformatics analysis.

### 2.1. Ox-PTMs and Highly Oxidized Proteins in the Cerebrum and Heart

**Figure 1:**
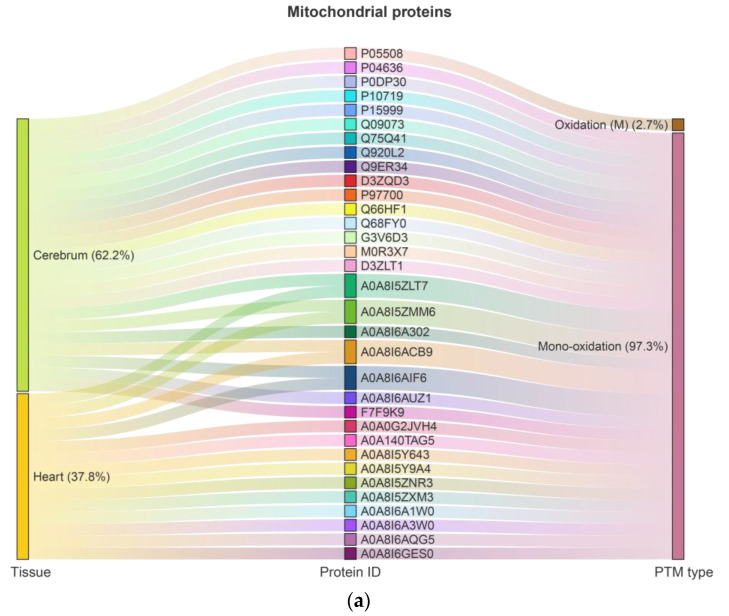

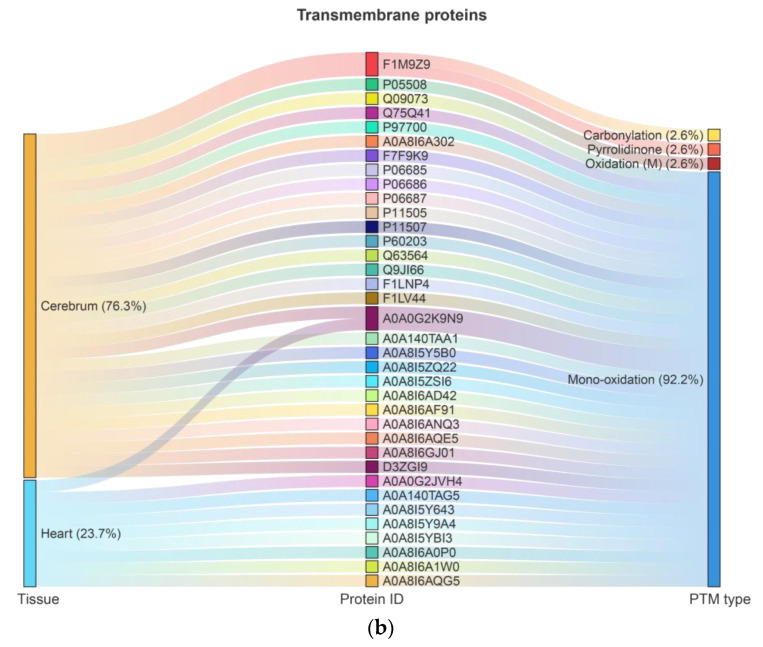
Distribution of highly oxidized proteins in rat cerebrum and heart: (**a**) mitochondrial proteins; (**b**) transmembrane proteins.

In the cerebrum and heart of rats, 977 and 366 proteins, respectively, were identified with up to six types of Ox-PTMs, including 2-amino-3-ketobutyric acid, kynurenine, pyrrolidinone, carbonylation, oxidation (M), and mono-oxidation (Tables S2 and S3). Among these, 139 proteins were consistently highly oxidized (oxidation rate ≥ 0.8) across all experimental groups, regardless of age or dietary factors. These proteins included 33 mitochondrial and 36 transmembrane proteins, with 12 proteins classified as both mitochondrial and transmembrane. Among mitochondrial proteins, 23 (62.2%) originated from the cerebrum and 14 (37.8%) from the heart, with 4 proteins jointly identified; oxidation (M) was observed on 1 protein (2.7%) and mono-oxidation on 32 proteins (97.3%) (Figure 1a, Table S4). Among transmembrane proteins, 29 (76.3%) originated from the cerebrum and 9 (23.7%) from the heart, with 2 proteins jointly identified; four types of Ox-PTMs were detected: carbonylation 1 (2.6%), pyrrolidinone 1 (2.6%), oxidation (M) 1 (2.6%), and mono-oxidation 33 (92.2%) (Figure 1b, Table S5).

Modification rates for each protein and Ox-PTMs in each group were summarized in Supplementary Tables S4 and S5 as Mean ± SD, calculated across the proteins within each category. For groups containing only a single protein, SD is not applicable (NA). No statistical comparisons between groups were performed because the primary aim was to identify proteins with high oxidation across all groups for subsequent bioinformatics analyses, rather than to assess differential effects between groups. Whether mitochondrial or transmembrane, the proportion of mono-oxidation was very high, reaching 92% to 97%. Many key mitochondrial proteins, including ETC complexes and ATP synthase subunits, are themselves transmembrane proteins; thus, classification as mitochondrial or transmembrane is not mutually exclusive, and the dual identity must be considered when interpreting oxidation patterns, network connectivity, and functional resilience.

### 2.2. GO Enrichment Analysis

**Figure 2:**
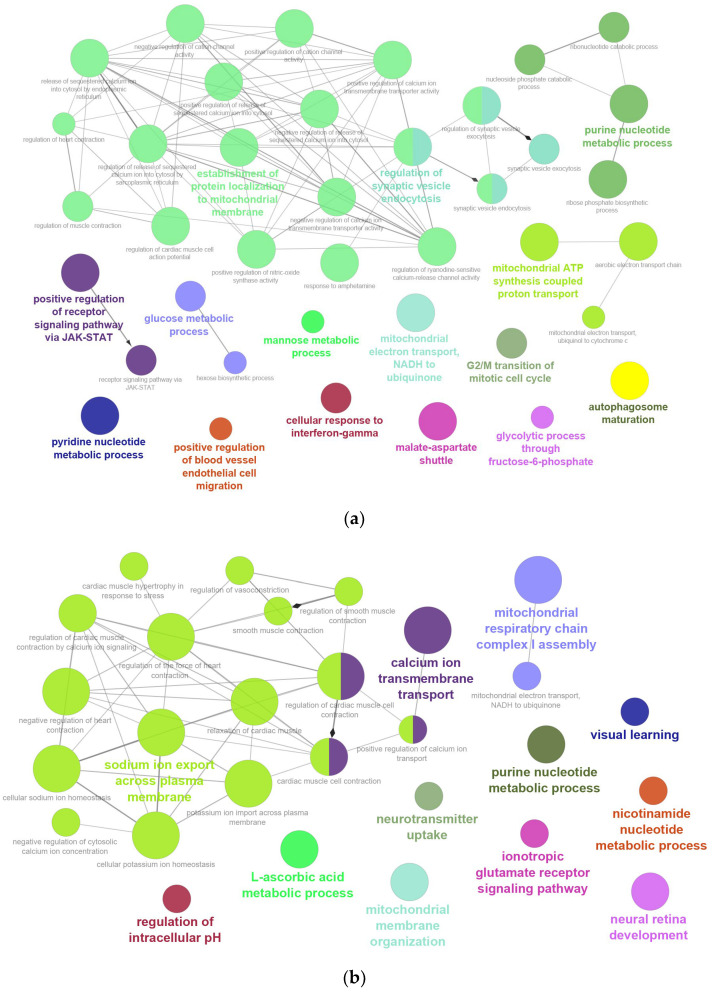
Gene Ontology (GO) enrichment analysis of biological processes (BP): (**a**) mitochondrial proteins; (**b**) transmembrane proteins.

Gene Ontology (GO) enrichment analysis of highly oxidized proteins was performed using ClusterProfiler and visualized with ClueGo (*p*.adjust < 0.05). GO analysis was interpreted across the three standard ontologies: Biological Process (BP), Cellular Component (CC), and Molecular Function (MF). BP analysis indicated that mitochondrial proteins were mainly involved in establishment of protein localization to mitochondrial membrane (42.86%), purine nucleotide metabolic process (9.52%), regulation of synaptic vesicle endocytosis (9.52%), mitochondrial ATP synthesis coupled proton transport (7.14%), carbohydrate metabolism including glucose and mannose metabolic processes (7.14%), mitochondrial electron transport and malate–aspartate shuttle (4.76%), glycolytic process through fructose-6-phosphate (2.38%), and pyridine nucleotide metabolic process (2.38%) (Figure 2a and Figure S1, Table S6). Transmembrane proteins were enriched in sodium ion export across plasma membrane (51.61%), calcium ion transmembrane transport (12.9%), mitochondrial respiratory chain complex I assembly (6.45%), mitochondrial membrane organization (3.23%), neurotransmitter uptake (3.23%), neural development and visual learning processes (6.46%), regulation of intracellular pH (3.23%), ionotropic glutamate receptor signaling (3.23%), and nucleotide metabolism including L-ascorbic acid and nicotinamide nucleotide processes (6.46%) (Figure 2b and Figure S2, Table S7).

**Figure 3:**
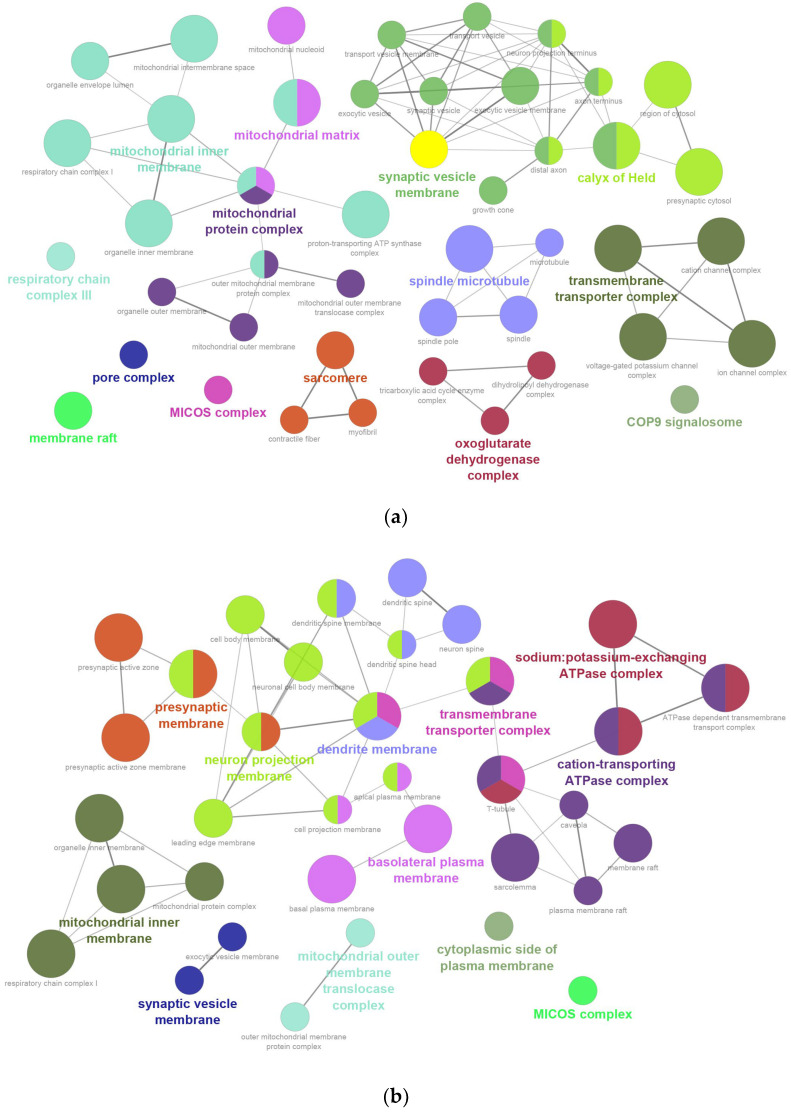
Gene Ontology (GO) enrichment analysis of cellular components (CC): (**a**) mitochondrial proteins; (**b**) transmembrane proteins.

CC analysis showed mitochondrial proteins were localized primarily to synaptic vesicle membrane (20.75%), mitochondrial inner membrane (16.98%), calyx of Held (11.32%), mitochondrial protein complexes (9.43%), transmembrane transporter complexes (7.55%), sarcomere and spindle microtubule (13.21%), mitochondrial matrix (5.66%), oxoglutarate dehydrogenase complex (5.66%), and other minor complexes (7.55%) (Figure 3a and Figure S3, Table S8). Transmembrane proteins were distributed across neuron projection membranes (22.45%), cation-transporting ATPase complexes (16.33%), dendrite and presynaptic membranes (18.36%), mitochondrial inner membrane (8.16%), basolateral plasma membrane (8.16%), sodium: potassium-exchanging ATPase complexes (8.16%), transmembrane transporter complexes (6.12%), mitochondrial outer membrane translocase complex (4.08%), synaptic vesicle membrane (4.08%), cytoplasmic side of plasma membrane (2.04%) and MICOS complex (2.04%) (Figure 3b and Figure S4, Table S9).

**Figure 4:**
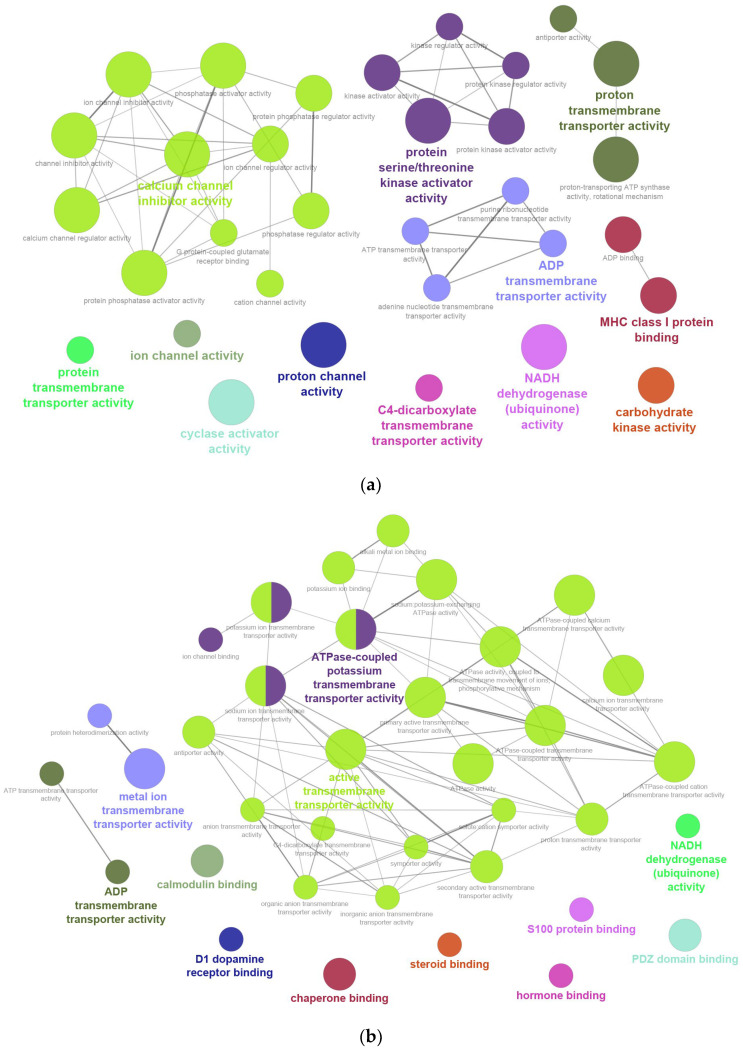
Gene Ontology (GO) enrichment analysis of molecular functions (MF): (**a**) mitochondrial proteins; (**b**) transmembrane proteins.

MF analysis revealed that mitochondrial proteins were mainly associated with calcium channel inhibitor activity (35.48%), protein kinase activator activity (16.13%), ADP and proton transmembrane transporter activities (22.58%), MHC class I protein binding (6.45%), and other transporter or enzyme activities (9.68%) (Figure 4a and Figure S5, Table S10). Transmembrane proteins were enriched in active transmembrane transporter activity (62.16%), ATPase-coupled potassium transport (10.81%), ADP transmembrane transporter activity (5.41%), NADH dehydrogenase activity (2.7%), and various binding activities including calmodulin, PDZ domain, dopamine receptor, steroid, hormone, S100 proteins, and chaperones (18.92%) (Figure 4b and Figure S6, Table S11).

### 2.3. Enrichment Pathway Network Analysis

**Figure 5:**
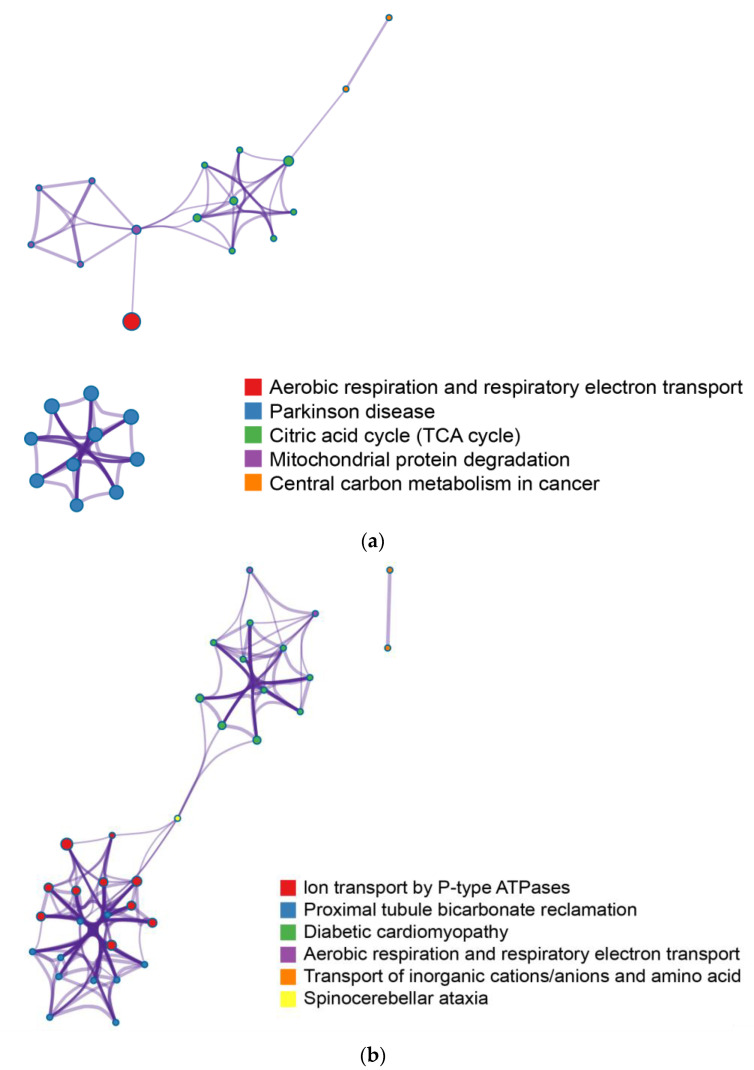
Pathway enrichment analysis of highly oxidized proteins: (**a**) mitochondrial proteins; (**b**) transmembrane proteins. Enrichment plots were generated using Metascape, which applies *p*-value ranking. Adjusted *p*-values (Benjamini–Hochberg FDR) are provided in Supplementary Table S6.

Based on Metascape analysis using multiple pathway databases, we performed an integrated pathway analysis of mitochondrial and transmembrane proteins. For mitochondrial proteins, 31 significant pathways (*p* < 0.01) were identified and grouped into five major functional categories: oxidative phosphorylation/respiratory electron transport, TCA cycle, Parkinson’s disease-related pathways, protein degradation, and central carbon metabolism (Figure 5a, Table S12). For transmembrane proteins, 47 significant pathways (*p* < 0.01) were identified and summarized into six categories: ion transport by P-type ATPases, aerobic respiration/respiratory electron transport, metabolite transport, proximal tubule bicarbonate reclamation, disease-related pathways (e.g., diabetic cardiomyopathy, spinocerebellar ataxia), and amino acid/peptide transport (Figure 5b, Table S13). This approach highlights functional clusters relevant to energy metabolism, ion homeostasis, and disease-associated processes.

### 2.4. PPI Network Construction

**Figure 6:**
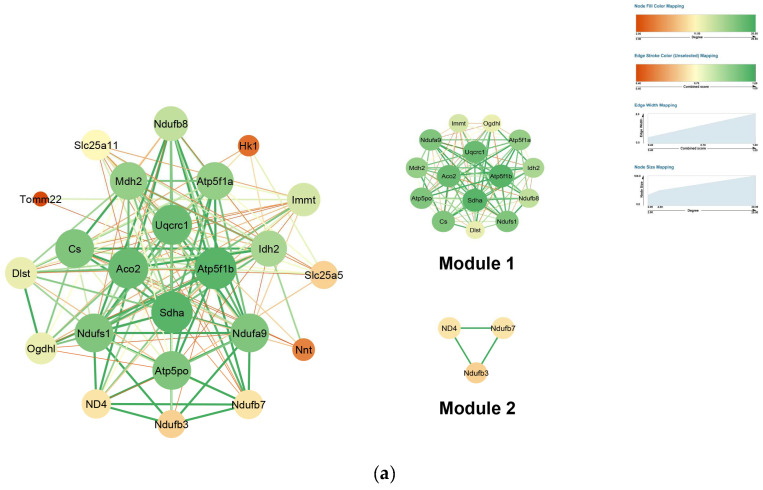

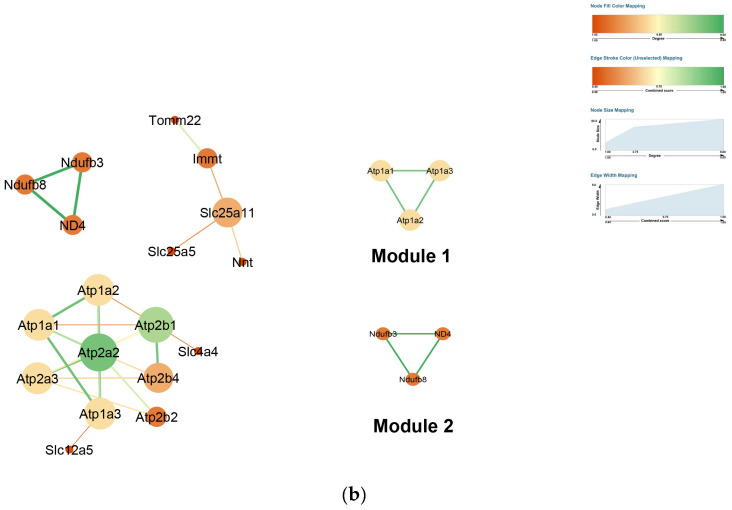
Protein–protein interaction (PPI) networks of highly oxidized proteins: (**a**) mitochondrial proteins; (**b**) transmembrane proteins.

Protein–protein interaction (PPI) networks of mitochondrial and transmembrane proteins were constructed using the STRING database and visualized in Cytoscape (v3.10.3). Hub proteins were defined based on node degree. In the mitochondrial network, top hubs included *SDHA*, *ACO2*, *ATP5F1B*, *UQCRC1*, *CS*, *NDUFA9*, *NDUFS1*, and *ATP5PO*. *ATP5F1B* exhibited the highest betweenness centrality (0.08), suggesting a key role in mediating communication between functional modules, while *TOMM22* showed the highest clustering coefficient (1.0), indicating tight connectivity with its interaction partners (Figure 6a, Table S14). In the transmembrane network, hubs included *ATP2A2*, *ATP2B1*, *ATP1A1/2/3*, and *ATP2A3*, with *SLC25A11* displaying the highest betweenness centrality (0.83) and *SLC25A11*, *IMMT*, and *ATP2A2* having the highest clustering coefficients (1.0) (Figure 6b, Table S15).

Module analysis using MCODE revealed distinct functional clusters. In mitochondria, Module 1 (score: 13.9) comprised TCA cycle enzymes (*Aco2*, *Mdh2*, *Idh2*, *Cs*, *Dlst*, *Ogdhl*) and subunits of respiratory complexes I–V (*Ndufs1*, *Ndufb8*, *Ndufa9*, *Sdha*, *Uqcrc1*, *Atp5f1a*, *Atp5f1b*, *Atp5po*), representing a cluster centered on energy metabolism and oxidative phosphorylation. Module 2 (score: 3.0) contained Complex I subunits *ND4*, *NDUFB3*, and *NDUFB7*, highlighting NADH dehydrogenase activity and electron transport. In transmembrane proteins, Module 1 (score: 3.0) included Na^+^/K^+^-ATPase α subunits (*Atp1a1*, *Atp1a2*, *Atp1a3*), emphasizing ion transport and excitability regulation, while Module 2 (score: 3.0) comprised *ND4*, *NDUFB3*, and *NDUFB8*, corresponding to mitochondrial Complex I and oxidative phosphorylation functions (Tables S16 and S17). Given the identification of oxidized subunits in key respiratory modules, we evaluated the enzymatic activity of Complexes I and IV to determine whether these oxidative modifications are accompanied by detectable functional changes.

### 2.5. In-Gel Activity of Mitochondrial Complexes I and IV in Cerebrum

**Figure 7:**
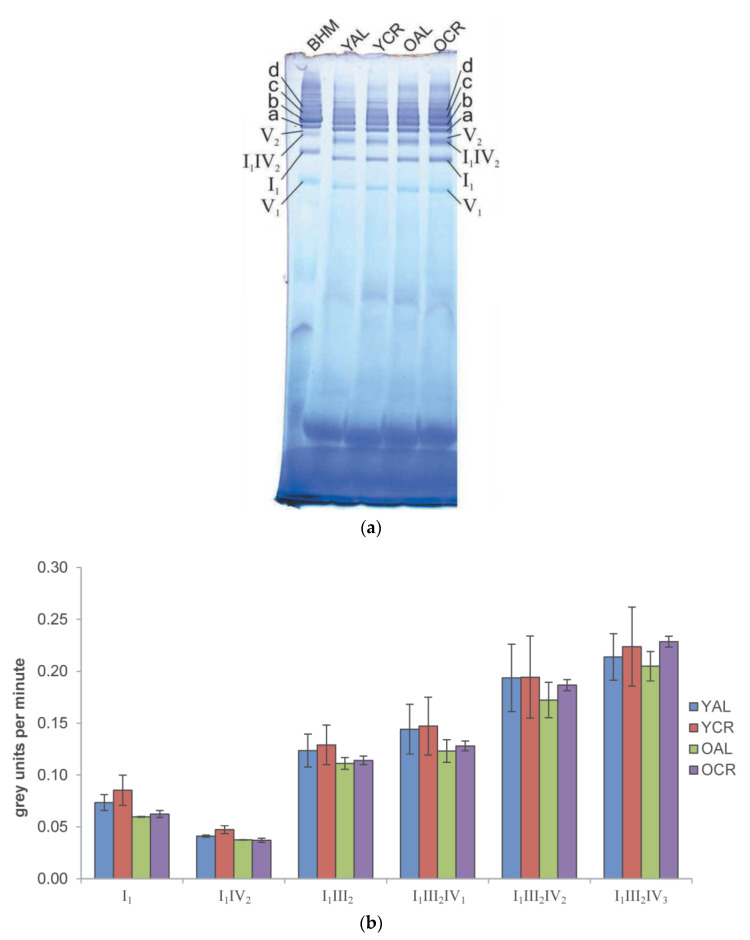
In-gel activity of mitochondrial Complex I in rat cerebrum: (**a**) Representative BN-PAGE gel showing in-gel activity staining for Complex I (CI). Mitochondrial proteins (100 µg) from each cerebrum sample group (YAL, YCR, OAL, and OCR) were separated. CI activity is visualized by blue/purple formazan precipitate. Key supercomplexes are annotated: a, I_1_III_2_; b, I_1_III_2_IV_1_; c, I_1_III_2_IV_2_; d, I_1_III_2_IV_3_. Bovine heart mitochondria (BHM, 70 µg) were included as a reference. (**b**) Quantitative analysis of CI-specific activity in individual complexes and supercomplexes. For each cerebrum animal group (YAL, YCR, OAL, OCR), the specific activity of CI in each analyzed gel band is plotted (in grey units per minute). Bar plots represent the mean of technical replicates (*n* = 3), with error bars showing the standard error of the mean.

To assess whether the persistent protein oxidation identified in our proteomic study affects mitochondrial function, Blue Native PAGE (BN-PAGE) in-gel activity assays were performed for Complex I (CI) and Complex IV (CIV) using cerebrum mitochondria. Notably, our proteomic and PPI analyses identified several subunits of CI (e.g., *NDUFA9*, *NDUFS1*) and CIV as being highly oxidized and residing within key functional modules. For Complex I, activity was visualized by blue/purple formazan formation, and well-defined bands corresponding to Complex I_1_ and various supercomplexes (I_1_IV_2_, I_1_III_2_, I_1_III_2_IV_1_, I_1_III_2_IV_2_, and I_1_III_2_IV_3_) were detected across all experimental groups (YAL, YCR, OAL, and OCR) (Figure 7a). Quantitative analysis revealed no statistically significant changes attributable to aging or CR in any complex or supercomplex (Figure 7b), indicating that oxidation of these specific CI subunits does not cause detectable loss of enzymatic activity.

**Figure 8:**
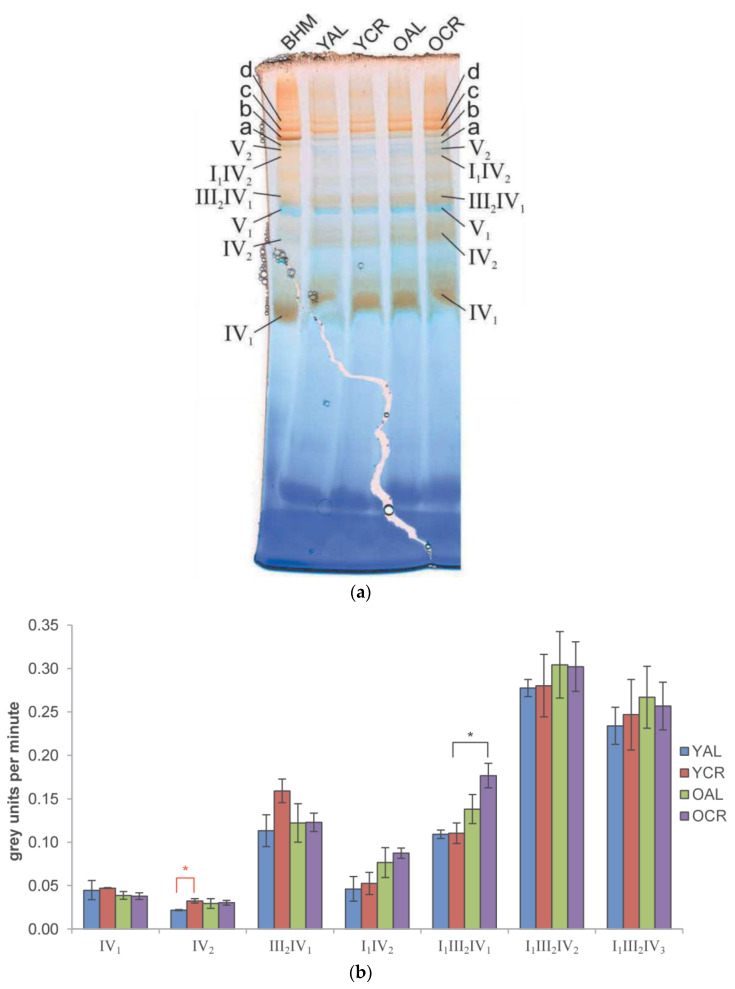
In-gel activity of mitochondrial Complex IV in rat cerebrum: (**a**) Representative BN-PAGE gel showing in-gel activity staining for Complex IV (CIV). Enzymatic activity is visualized by brownish DAB precipitate. a, I_1_III_2_; b, I_1_III_2_IV_1_; c, I_1_III_2_IV_2_; d, I_1_III_2_IV_3_. Sample loading and reference standards were identical to those used for CI. (**b**) Quantitative analysis of CIV-specific activity in homooligomeric and supercomplex-associated forms. For each cerebrum animal group (YAL, YCR, OAL, and OCR), the specific activity of CIV in each analyzed gel band is plotted (in grey units per minute). Bar plots show the mean of technical replicates (*n* = 3), with error bars representing the standard error of the mean. Statistical significance: * *p* < 0.05; black asterisk indicate changes caused by aging (YAL vs. OAL; YCR vs. OCR), and red asterisk indicate changes caused by CR (YAL vs. YCR; OAL vs. OCR).

By contrast, CIV in-gel activity assays showed distinct monomeric (IV_1_), dimeric (IV_2_), and supercomplex-associated forms (Figure 8a). Statistically significant enhancements in CIV activity were observed in response to dietary and age interventions (Figure 8b) only for two supercomplexes. Short-term CR (YAL vs. YCR) significantly increased the activity of the CIV dimer (IV_2_). Furthermore, within the cohort of calorie-restricted animals, aging itself was associated with a significant increase in CIV activity within the I_1_III_2_IV_1_ supercomplex (YCR vs. OCR). The activity of CIV within higher-order supercomplexes (e.g., I_1_III_2_IV_1_, I_1_III_2_IV_2_) was typically higher than in its monomeric form, suggesting a functional advantage of supercomplex assembly. No consistent age-related decline in CIV activity was observed under either dietary regime. Collectively, these results demonstrate that not only are the enzymatic functions of key mitochondrial respiratory complexes in the cerebrum preserved despite significant oxidation of core subunits, but they can even be enhanced under specific conditions of CR and within the context of aging, reflecting a profound functional tolerance and adaptive capacity to persistent oxidation.

### 2.6. Structural Integrity of Respiratory Complexes in Heart Mitochondria

**Figure 9:**
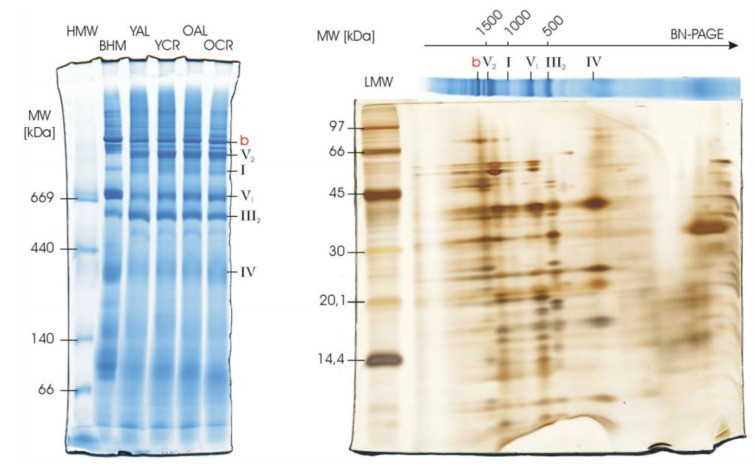
Blue Native PAGE analysis of respiratory complexes and supercomplexes (b: I_1_III_2_IV_1_) in heart mitochondria. Mitochondrial proteins from interfibrillar mitochondria (IFM) isolated from the hearts of young (Y) and old (O) rats under ad libitum (AL) or calorie-restricted (CR) regimes were separated by BN-PAGE. Mitochondria were solubilized with digitonin at a detergent-to-protein ratio of 4 g/g, and 80 µg of protein was loaded per lane. Individual respiratory complexes are labeled in black, and supercomplexes are labeled in red. The Coomassie-stained gel images show highly consistent banding patterns across all experimental groups, indicating that the structural assembly of respiratory complexes and supercomplexes is not overtly affected by aging or CR. The isolation of IFM required bacterial protease treatment, which led to partial protein degradation detectable in the second dimension of 2D-BN/SDS-PAGE (silver stained) but did not compromise the resolution of high-molecular-weight complexes in this first-dimension BN-PAGE. IFM samples were pooled within each experimental group for analysis due to limited mitochondrial yield.

We next examined whether persistent oxidation affects the structural assembly of oxidative phosphorylation complexes in the heart, particularly given the identification of oxidized structural subunits such as *UQCRC1* (Complex III) and *ATP5F1B* (ATP synthase) in our PPI network. Blue Native PAGE (BN-PAGE) analysis was performed on interfibrillar mitochondria (IFM). Well-resolved bands corresponding to individual respiratory complexes and their supercomplexes were observed across all experimental groups (Figure 9). Critically, the banding patterns in Coomassie-stained BN gels were highly consistent, revealing no overt differences in the assembly or abundance of these high-molecular-weight complexes between young and aged animals, or between ad libitum and calorie-restricted groups.

It is noteworthy that the isolation of IFM required bacterial protease treatment, which resulted in partial protein degradation, as evidenced in the second dimension of 2D-BN/SDS-PAGE (Figure 9). However, this degradation primarily affected lower molecular weight proteins and did not compromise the integrity or resolution of the native complexes and supercomplexes in the first-dimension BN-PAGE, which remains a robust measure of structural assembly. Furthermore, samples for IFM analysis were pooled within experimental groups due to limited mitochondrial yield. Together, these BN-PAGE findings, along with the preserved enzymatic activities in the cerebrum, provide strong evidence that the identified oxidation burden does not disrupt the structural integrity or functional organization of the mitochondrial respiratory system.

## 3. Discussion

Our study demonstrates that, despite widespread oxidative modification across mitochondrial and transmembrane proteins, many targets exhibit persistent high amounts of mono-oxidation without measurable responsiveness to aging and CR, and without larger detrimental effects on the structure and function of two key protein complexes of the respiratory chain. This distinction suggests that cells have evolved a combination of structural and functional tolerance mechanisms, including molecular and network-level strategies, allowing essential proteins and networks to maintain metabolic robustness even under chronic ROS exposure. Importantly, these observations reveal that oxidative marks cannot be universally interpreted as indicators of dysfunction, and their impact depends on residue localization, stoichiometry, and protein context. This insight provides a framework for understanding the dual role of ROS in mitochondria as both a damaging agent and a signaling mediator, while many proteins and networks tolerate persistent mono-oxidation, and sets the stage for exploring the molecular and network-level strategies underlying cellular resilience.

### 3.1. ROS and Protein Oxidation: Background and Functional Implications

ROS are unavoidable by-products of aerobic metabolism, predominantly generated in mitochondria through the electron transport chain (ETC) during oxidative phosphorylation [19], and can lead to various forms of protein oxidation. These chemical changes can impair protein folding, enzymatic activity, and interactions, but not all oxidative events necessarily result in functional inactivation [43]; in fact, they are sometimes important signal transduction effectors [44,45].

Baseline measurements indicate that in young mammalian tissues, mitochondrial protein carbonyl levels typically range from approximately 0.4 to 1.0 nmol of carbonyl groups per mg of protein, with methionine oxidation occupying around 1–3% of target residues [46], suggesting that, under normal physiological conditions, cells can efficiently manage oxidative modifications through repair and turnover systems [47]. In our dataset, we identified 139 proteins exhibiting consistently elevated oxidation (ratio ≥ 0.8), including 23 and 14 proteins from cerebrum and heart mitochondria, respectively, and 29 and 9 proteins from cerebrum and heart transmembrane compartments. Mono-oxidation accounted for 92–97% of these high-oxidation proteins, whereas other modifications such as carbonylation or pyrrolidinone formation were much less frequent (Figure 1a,b). The persistent mono-oxidation appeared largely independent of age or CR, indicating widespread structural or functional tolerance rather than selective repair.

Proteomic surveys across tissues and species consistently identify mitochondrial enzymes, ETC complexes, ATP synthase subunits, and cytoskeletal/membrane proteins as frequent oxidation targets [48,49]. Quantitative analyses show that oxidation stoichiometry is often low (<10% of total molecules) yet disproportionately impactful when it affects catalytic residues or metal clusters [22,50]. It has been reported previously that Ox-PTMs occurred at surface residues or redundant structural domains, allowing accumulation without measurable functional decline [51]. Our results align with this broader picture: we observed widespread oxidative modification across mitochondrial and transmembrane proteins, many of which are canonical targets reported in aging and stress models [10]. However, a subset of proteins showed persistently high oxidation without age or CR responsiveness, consistent with intrinsic protein tolerance. These findings suggest that oxidative modifications should be interpreted based on residue localization, stoichiometry, and functional context, rather than assumed to indicate universal damage.

### 3.2. ROS Management and Resilience in Aging and CR

Aging is strongly linked to oxidative stress, with studies showing increased levels of protein carbonylation, cysteine overoxidation, and tyrosine nitration in aged tissues [52,53,54]. Many of these changes are associated with loss of activity in enzymes central to energy metabolism and signaling, supporting the classical oxidative stress theory of aging [13]. CR, one of the most robust lifespan-extending interventions, has repeatedly been shown to lower mitochondrial ROS production, preserve Fe–S clusters, and maintain redox homeostasis [34,55,56]. These effects are mediated by improved mitochondrial efficiency, induction of antioxidant enzymes, and enhancement of quality control mechanisms such as mitophagy and unfolded protein responses [57,58].

Important functional evidence for resilience in our study comes from the CIV in-gel activity assays. Contrary to the notion that oxidation inevitably leads to decline, we observed a significant enhancement of CIV activity in specific contexts: upon short-term CR in the dimeric form (IV_2_), and with aging within the I_1_III_2_IV_1_ supercomplex in CR animals. These findings suggest that the system is not merely passive in the face of oxidative stress but can actively adapt (Figure 8a,b). This enhancement aligns with studies showing that CR and metabolic health are associated with optimized respiratory chain organization, including the stabilization of supercomplexes, which can improve electron transfer efficiency and reduce electron leak, thereby potentially increasing catalytic activity [56]. This demonstrates a capacity for functional enhancement that operates independently of, or even concurrently with, the persistent oxidative modifications we identified.

In our dataset, a substantial number of mitochondrial and membrane-associated proteins—including ATP synthase subunits (*ATP5PO*, *Atp5f1b*), Complex I accessory proteins (*NDUFA9*, *NDUFS1*, *NDUFB3/B7/B8*), TCA cycle enzymes (*Sdha*, *Aco2*, *Cs*), Na^+^/K^+^-ATPase α/β subunits, *Slc25a11*, *SNAP25*, *VAMP2*, and large structural proteins such as *MYH* isoforms and spectrin chains—remained highly oxidized regardless of age or CR (Figure 6). This indicates widespread intrinsic tolerance to persistent mono-oxidation, consistent with previous reports: *ATP5PO* oxidation has been documented in aged rat hearts without measurable loss of ATP synthase activity [59]; Complex I accessory subunits can accumulate oxidative marks without impairing assembly [60,61]; and heavily oxidized cytoskeletal proteins retain structural integrity due to redundancy and polymeric organization [8,62].

Cells also possess repair and turnover systems, including methionine sulfoxide reductases, thioredoxin/glutaredoxin systems, and selective mitophagy, which help contain the impact of oxidative lesions [63,64]. Functional redundancy in protein families—such as multiple isoforms of Na^+^/K^+^-ATPase subunits or TCA cycle enzymes—provides buffering capacity against oxidative insults, while sacrificial or “decoy” residues may preferentially absorb oxidative stress, protecting more sensitive catalytic cores [65]. Taken together, these mechanisms reveal a dual strategy for coping with oxidative stress: CR and other interventions reduce ROS to protect vulnerable catalytic hubs, while many proteins have evolved intrinsic tolerance that allows them to function despite persistent mono-oxidation at one or more amino acid residues. This resilience is demonstrated by the significant enhancements in CIV activity in the cerebrum (Figure 8b) and the preserved structural integrity of respiratory supercomplexes in the heart (Figure 9) observed in our study. The efficacy of this protective strategy is underscored by its stark contrast to the mitochondrial dysfunction and amplified oxidative damage observed in settings of metabolic overload, such as obesity [66,67].

It is worth mentioning that while our experimental design was not aimed at studying the effect of obesity, under AL, the weight of animals significantly increased over time. Therefore, one can speculate that the persistent and abundant Ox-PTMs detected in our study have functional implications for obesity, too.

### 3.3. Network-Level Resilience and a Dual Evolutionary Strategy

Our enrichment and network analyses revealed that oxidation tends to cluster within specific functional modules: aerobic respiration and the TCA cycle in mitochondria, and excitation–contraction coupling, vesicle trafficking, and structural proteins in membranes (Figure 5 and Figure 6). Within these modules, certain hub proteins—including *SDHA*, *ACO2*, *ATP5F1B*, *UQCRC1*, *CS*, *ATP5PO* in mitochondria, and Na^+^/K^+^-ATPase α/β subunits, *Slc25a11*, *SNAP25*, *VAMP2*, and *MYH* isoforms in membrane systems—exhibited high centrality, suggesting that their modification could potentially destabilize entire networks (Figure 6). Notably, despite persistent mono-oxidation, several of the hub proteins detected here (e.g., *SDHA*, *ACO2*, *ATP5F1B*/*ATP5PO*, *UQCRC1*, and *CS*) have been repeatedly observed as oxidized in proteomic surveys while functional studies or orthogonal assays report preserved overall respiratory/enzymatic activity under comparable conditions [39,68,69]. In line with this, our BN-PAGE analysis of heart mitochondria revealed that the oxidative modifications identified on key structural hubs like *UQCRC1* and *ATP5F1B* did not translate into macroscopic defects in the assembly of respiratory complexes or supercomplexes (Figure 9). This indicates that cells are not merely avoiding oxidation at the molecular level but are organized in a way that allows essential processes to persist even when key proteins are chemically modified, according to experimental evidence from our data.

The modular organization of protein interaction networks adds an additional layer of robustness. Redundant pathways, compensatory fluxes, and distributed control mechanisms can buffer the effects of localized oxidative stress, preventing the collapse of essential metabolic or signaling networks [70]. For example, persistent oxidation in mitochondrial Complex I accessory subunits does not compromise overall respiration, as central catalytic subunits remain protected, repaired, or complemented by isoforms [68], in accordance with our data. Similarly, membrane and synaptic hubs (Na^+^/K^+^-ATPase α/β, *Slc25a11*, *SNAP25*, *VAMP2*, *MYH* isoforms) are recurrent oxidation hits in tissue proteomics but have been shown in prior studies to retain core activity or structural integrity in several models [71,72,73]. These network-level observations emphasize that resilience is not solely a property of individual proteins, but emerges from the integration of multiple layers of regulation, redundancy, and repair [74].

Taken together, our data support the view that cells employ a multi-tiered, dual evolutionary strategy to cope with oxidative stress [16]. At the molecular level, ROS production is minimized and antioxidant defenses are enhanced—exemplified by interventions such as CR and mitochondrial quality control pathways—to protect vulnerable catalytic cores [17]. Modulation of oxidative phosphorylation machinery allows for maintenance of its activity and glutathione redox status [75,76]. Simultaneously, proteins and networks exhibit intrinsic tolerance to oxidation through structural features, redundancy, and repair systems, allowing persistent modifications in less critical or structurally buffered components without compromising essential functions [77]. This integrated, systems-level perspective aligns with evolutionary principles of robustness, where redundancy and modularity safeguard cellular integrity even under chronic oxidative pressure [78].

### 3.4. Limitations and Translational Considerations

This study has several limitations. First, the proteomic analyses were based on a relatively small sample size (*n* = 3 biological replicates per group for LC–MS/MS), which may limit statistical power for detecting subtle group differences. Second, our focus on proteins with consistently high oxidation ratios (≥0.8) provided insights into highly oxidized targets but excluded moderately oxidized proteins that may also contribute to redox regulation and aging phenotypes. Third, while the BN-PAGE in-gel activity and assembly analyses demonstrated that persistent oxidation does not impair Complex I and IV functionality or structural integrity, additional biochemical and genetic validation will be necessary to fully determine the physiological consequences of persistent mono-oxidation.

Beyond these technical considerations, other oxidative protein modifications—such as lipoxidation or nitration—were not examined in this study, even though they represent important mechanisms of redox regulation and damage. Additionally, oxidative stress can induce protein cross-linking, often secondary to lipid peroxidation. Lipoxidation, in particular, involves covalent modification by reactive lipid peroxidation products such as 4-hydroxynonenal (4-HNE) and malondialdehyde (MDA), which have been implicated in enzyme inactivation, membrane disruption, and age-related as well as inflammatory diseases [79,80,81]. While lipoxidation was not directly assessed in our dataset, its potential occurrence in similar redox environments may provide complementary insight into oxidative signaling and damage. Incorporating these less abundant PTMs, along with an assessment of protein cross-linking, into future redox-proteomic workflows could provide a more comprehensive understanding of oxidative stress adaptation and help distinguish between tolerable and pathological oxidation.

To place our findings in a broader context, previous proteomic studies in aged rodent tissues have also reported widespread mono-oxidation of mitochondrial and cytoskeletal proteins without detectable loss of enzymatic activity or metabolic output [28,82,83]. This consistency supports the view that persistent mono-oxidation often reflects intrinsic structural tolerance rather than dysfunction. Although our experiments were performed in rats, the dominance of mono-oxidation and the resilience of mitochondrial and transmembrane proteins may represent conserved features across species. Future integrative approaches combining redox proteomics, functional assays, and oxidative lipid-adduct mapping will be essential to validate these mechanisms and assess their relevance to human aging and metabolic disorders.

## 4. Materials and Methods

### 4.1. Animal Experiments and Mitochondrial Sample Collection

Male F344 Fischer rats (*Rattus norvegicus*) were purchased from Charles River, Tokyo, Japan, and maintained under specific pathogen-free (SPF) conditions at the Tokyo Metropolitan Institute of Geriatrics and Gerontology (TMIGG). The experimental protocol was approved by the Ethics Review Committee for Animal Experimentation of TMIGG, Japan. At 4 wk of age, weanling male rats were transferred to a barrier facility (temperature 22–25 °C, 12 h light/dark cycle), housed separately, with ad libitum access to water. Environmental enrichment included standard nesting material and shelters. The study design comprised four conditions: young (Y) and old (O) animals under ad libitum (AL) or calorie-restricted (CR). The experimental unit was the individual animal. Animals were either fed AL or subjected to CR starting from week 6 after birth for either short-term (6.5 months) or long-term (27 months) intervention. CR animals were fed AL on three days per week (Mondays, Wednesdays, and Fridays; alternate day feeding), resulting in food intake of approximately 60% of AL levels (CR levels: 40% for young, 37% for old animals). In total, 22 rats were included: YAL (*n* = 5), YCR (*n* = 5), OAL (*n* = 6), and OCR (*n* = 6). Body weights of all animals at the time of sacrifice were recorded (Table S1). No formal randomization was applied to allocate animals to experimental groups.

For proteomic LC–MS/MS analysis, three biological replicates were selected from each group (*n* = 3 per group), while the remaining animals were used for additional biochemical and validation experiments. The sample size for proteomics was based on prior proteomic studies demonstrating sufficient reproducibility. Treatments and sample collection were performed on the same day for all animals. Investigators were not blinded to group allocation during experiments and data collection.

Following sacrifice, frozen tissue samples (heart and cerebrum) were transported to Germany on dry ice. Mitochondria were isolated separately from the heart and cerebrum tissues of each animal, and proteomic analyses were performed independently for each tissue type without pooling between organs. Isolation was conducted at 4 °C following the procedure of Johnson and Lardy [84]. Briefly, freshly dissected tissues were rinsed in ice-cold isolation buffer A (50 mM Tris, pH 7.4, 200 mM mannitol, 50 mM sucrose, 1 mM EDTA, 5 mM EGTA, supplemented with antioxidants and protease inhibitor), and homogenized using a tight-fit glass/Teflon homogenizer (B. Braun Biotech International, Melsungen, Germany) (4–5 strokes at 800 rpm) on ice. The homogenate was centrifuged at 80× *g* for 10 min to remove nuclei and cell debris, and the resulting supernatant was layered on buffer B (identical to buffer A but containing 150 mM sucrose) and centrifuged at 700× *g* for 10 min. The upper supernatant was collected and centrifuged at 10,000× *g* for 10 min to obtain the crude mitochondrial pellet, which was washed twice with isolation buffer A and finally resuspended in storage buffer C (50 mM Tris, 200 mM mannitol, 50 mM sucrose, and protease inhibitor cocktail). Protein concentrations were determined using the Bradford assay. For downstream proteomic analysis, only proteins annotated with mitochondrial subcellular localization were retained.

### 4.2. Extraction, Digestion, and Labeling of Proteins

Processing of mitochondrial samples using a modified in-filter protein digestion (FASP) procedure and tryptic digestion was performed according to C. Ramallo et al. [29]. All mitochondrial samples from each tissue sample were mixed in equal proportions to create an iTRAQ-labeled mix that served as a spike-in internal standard. A 4-plex iTRAQ kit was used for relative quantification, and therefore, three biological replicates of each condition were labeled with the remaining iTRAQ reagents as described previously [29].

### 4.3. LC-MS/MS Analysis

Samples were resuspended in 2% ACN in 0.1% FA to a final concentration of 1.5 μg/μL by sonication for 10 min before one-dimensional nLC-ESI-MS/MS analysis. The measurements were performed on an LTQ-Orbitrap Elite mass spectrometer (Thermo Fisher Scientific, Bremen, Germany) coupled to a nanoACQUITY gradient UPLC pump system (Waters, Milford, MA, USA). The peptides were separated in the same manner using reversed-phase chromatography as described in C. Ramallo et al. [29]. However, 5 μL of the sample was loaded directly onto the 25 cm analytical column using the nanoACQUITY autosampler (Waters), the column oven was set to 55 °C, and the reequilibration of the column was extended by 20 min.

Data-dependent acquisition on the LTQ-Orbitrap Elite was operated via instrument method files of Xcalibur (Rev. 2.1.0) in positive ion mode at a spray voltage of 1.8 kV. Methods using exclusively HCD fragmentation when acquiring MS/MS spectra consisted of an Orbitrap full MS scan followed by up to 20 HCD-Orbitrap MS/MS spectra (Top20, Bottom20; explanation see C. Ramallo et al. [29]) on the most abundant ions detected in the full MS scan. The full MS scan was performed in the Orbitrap in the range of 150–2000 m/z at a resolution of 120,000. The AGC for full MS experiments was set to 1 × 10^6^ with a maximum ion injection time of 500 ms. For peptide identification and reporter ion quantification, HCD fragmentation spectra were acquired with a normalized collision energy of 35%, isolation width of 1.5 Th, and activation time of 0.1 ms at a resolution of 15,000. The AGC for the MS/MS experiments was set to 1 × 10^4^ at a maximum ion accumulation time of 250 ms. Dynamic exclusion was enabled with a repeat count of one and a 45 s exclusion duration window. Unassigned charge states and singly charged ions were rejected from MS/MS. For each 4-plex sample, 4 technical replicates were performed, i.e., 2 × Top20 and 2 × Bottom20.

### 4.4. BN-PAGE, In-Gel Activity Assays, and Silver Staining

Mitochondrial samples from cerebrum and heart tissues were solubilized with digitonin at optimized detergent-to-protein ratios (4 g/g for heart; 8 g/g and 3 g/g for cerebrum, depending on experimental requirements, as optimized previously [85]) and separated by Blue Native PAGE (BN-PAGE) following established protocols as previously described [85]. For in-gel activity assays, cerebrum samples were incubated with activity-specific staining solutions: Complex I activity was detected using NADH and nitrotetrazolium blue (NBT), and Complex IV activity was visualized using diaminobenzidine (DAB) with cytochrome c according to established BN-PAGE activity staining methods [85].

For heart mitochondrial samples, 80 µg of protein was loaded per lane, and Coomassie Brilliant Blue staining was used to visualize the structural organization and relative abundance of respiratory complexes and supercomplexes. To further evaluate subunit integrity, two-dimensional BN/SDS-PAGE was performed for selected interfibrillar (IFM) mitochondrial samples. After second-dimension separation, gels were subjected to silver staining following the protocol of Blum et al. [86]. This enhanced-sensitivity staining method revealed partial degradation of lower-molecular-weight subunits, consistent with bacterial protease treatment during IFM isolation, while preserving the resolution of native complexes observed in the first BN dimension.

Gel images were captured and analyzed using ImageJ (v1.43) software, and band intensities were quantified to determine specific activities in grey units per minute. All assays were performed in technical triplicate (*n* = 3), and results are expressed as mean ± standard error.

### 4.5. Data Analysis

Protein identification and quantification in this iTRAQ experiment were performed with MaxQuant software (v2.1.3.0) using the implemented Andromeda search algorithm [87]. All acquired RAW files from one tissue were processed in one database search using MaxQuant software and were searched against the *Rattus norvegicus* protein database downloaded from http://www.uniprot.org (UP000002494; accessed on 17 August 2022) with 37,919 protein sequence entries, complemented by a database of common protein contaminants according to the standard Andromeda settings. Searches were performed using trypsin and LysC specificity, allowing 2 missed cleavages. Peptides with at least 6 amino acids were considered for identification while allowing LysC N-terminal cleavage to proline. Identification across different replicates was achieved by enabling the matching between runs option in MaxQuant within a time window of 0.7 min.

The precursor-ion mass tolerance was set to 20 ppm for the first search and 4.5 ppm for the main search. The fragment ion mass tolerance for HCD MS/MS spectra was kept by default of 20 ppm. The false discovery rate, determined by searching a reversed database, was set at 0.01 for both peptides and proteins. The analysis type in MaxQuant was set to Reporter ion MS2, and 4-plex iTRAQ was selected with a reporter mass tolerance of 0.01 Da. Filtering by precursor intensity fraction of >0.75 was enabled to avoid unwanted quantification of co-isolated peptides, and the identification of second peptides was disabled. Acetylation of the protein N-terminus and pyro-glutamic acid at peptide N-terminal glutamine residues were set as variable modifications. Additionally, oxidative modifications were considered as variable modification sites and were quantified consecutively in 2 database searches as indicated in Table S18. The minimum Andromeda score values for MS/MS identification of modified peptides were maintained by default (Min. score: 40; Min delta score: 6). Site localization probabilities were determined by MaxQuant using the PTM scoring algorithm, as described by Olsen et al. [88] and Cox & Mann [89].

### 4.6. Bioinformatics Analysis

Primary and secondary outcome measures were defined as follows: the primary outcome measure of this study was the oxidation degree of mitochondrial proteins across experimental groups. Secondary outcome measures included functional enrichment analyses of the identified proteins, such as Gene Ontology (GO) enrichment, pathway analysis, and protein–protein interaction (PPI) network characterization. Based on the number of modified and unmodified peptides identified in each group (YAL, OAL, YCR, and OCR) for each oxidative post-translational modification of each protein, the oxidation degree of each group (total number of modified peptides/total number of modified peptides + total number of unmodified peptides) was calculated. Then, the proteins with an oxidation rate of ≥0.8 in each group were summarized. Transmembrane proteins were filtered using the keywords “transmembrane” and acknowledged mitochondrial proteins were filtered using the gene ontology cellular component (GOCC) with terms containing “mitochondri” after the addition of the UniProt annotation of Rattus norvegicus in Perseus (v2.0.11) [90].

GO enrichment analysis of the identified proteins was performed in R (v4.5.1) using the clusterProfiler package (v4.16.0) [91]. Terms with *p*.adjust < 0.05 (Benjamini–Hochberg correction) were considered significant. GO terms were interpreted across three ontologies: Biological Process (BP), describing the biological programs or pathways in which proteins participate; Molecular Function (MF), referring to the biochemical activities of proteins at the molecular level; and Cellular Component (CC), denoting the subcellular locations or structural contexts where proteins are active. Pathway enrichment analysis was performed using the Metascape online platform (https://metascape.org; accessed on 3 August 2025) [92] with default parameters, integrating KEGG, Reactome, WikiPathways, and PANTHER pathway databases. In accordance with the platform’s default visualization settings, enriched pathways were plotted at a significance threshold of *p* < 0.01. For consistency across analyses, results were also evaluated using Benjamini–Hochberg adjusted *p*-values, and pathways with adjusted *p* < 0.05 were considered statistically significant. Protein–protein interaction (PPI) networks were generated using the STRING database (v12.0; https://string-db.org (accessed on 3 August 2025)) [93] with a confidence score cutoff of 0.4, and visualized in Cytoscape (v3.10.3) [94]. Network clustering was performed using the MCODE plugin (v2.0.3) [95] in Cytoscape to identify highly interconnected modules. Statistical analyses for enrichment and network clustering were conducted using the default algorithms of the respective software platforms (clusterProfiler, Metascape, STRING, and Cytoscape). A separate PCA was conducted to visualize the combined oxidative profile of cerebrum and heart tissues. For this analysis, we focused on three Ox-PTMs (mono-oxidation, carbonylation, and oxidation (M)) that provided robust and consistent spectral counts across the dataset, allowing for a reliable integrated analysis. The input data consisted of the normalized spectral counts for each of these three modifications, aggregated per sample. Data from both tissues were merged into a single matrix to enable direct comparison. The combined data matrix was logit-transformed prior to analysis to stabilize variance. PCA was conducted using R (v4.5.1) with centering and scaling enabled.

## 5. Conclusions

Our integrated proteomic and functional study reveals that a core set of mitochondrial and transmembrane proteins sustains persistent high oxidation, largely independent of age or caloric restriction. Crucially, this oxidative burden does not equate to functional decline but is compatible with preserved and even enhanced enzymatic activity in key respiratory complexes. This functional resilience, observed alongside maintained structural integrity, demonstrates a dual cellular strategy for oxidative stress management: proactive reduction in ROS is complemented by an inherent, multi-level robustness that confers not merely tolerance, but a capacity for functional adaptation to persistent oxidation, thereby ensuring metabolic homeostasis during aging.

## Data Availability

The mass spectrometry proteomics data have been deposited to the iProX repository (iProX ID: IPX0013383002) and will be publicly available upon acceptance of the manuscript.

## Supplementary Materials

The following supporting information is available online: https://www.mdpi.com/article/10.3390/ijms262211155/s1.

## Abbreviations

The following abbreviations are used in this manuscript:

ACN	Acetonitrile
AGC	Automatic Gain Control
AL	Ad Libitum
ANT	Adenine Nucleotide Translocator
BP	Biological Process
CC	Cellular Component
CR	Calorie Restriction
ETC	Electron Transport Chain
FA	Formic Acid
HCD	Higher-Energy Collisional Dissociation
IFM	Interfibrillar Mitochondria
iTRAQ	Isobaric Tags for Relative and Absolute Quantitation
LC-MS/MS	Liquid Chromatography–Tandem Mass Spectrometry
MF	Molecular Function
MFN2	Mitofusin-2
MCODE	Molecular Complex Detection
OCR	Old Calorie Restriction
OAL	Old Ad Libitum
OPA1	Optic Atrophy 1
Ox-PTMs	Oxidative Post-Translational Modifications
PCA	Principal Component Analysis
PPI	Protein–Protein Interaction
ROMO1	Reactive Oxygen Species Modulator 1
ROS	Reactive Oxygen Species
SDH	Succinate Dehydrogenase
SPF	Specific Pathogen-Free
STRING	Search Tool for the Retrieval of Interacting Genes/Proteins
TCA	Tricarboxylic Acid Cycle
TMIGG	Tokyo Metropolitan Institute of Geriatrics and Gerontology
VDAC	Voltage-Dependent Anion Channel
YCR	Young Calorie Restricted
YAL	Young Ad Libitum
