# Supplementary material for: Persistent Oxidation of Mitochondrial and Transmembrane Proteins in Rat Cerebrum and Heart Regardless of Age or Nutrition
Source: Int J Mol Sci. 2025 Nov 18;26(22):11155. doi: 10.3390/ijms262211155 (PMC12653704; doi:10.3390/ijms262211155)
Supplement: Supplementary file 1 [file ijms-26-11155-s001.zip › Supplementary materials.pdf]

Figure S1. Proportions of enriched biological process (BP) GO terms among highly oxidized mitochondrial proteins.

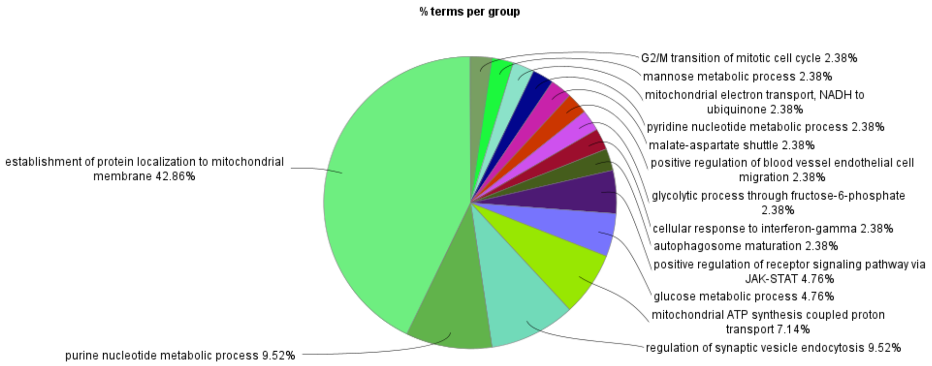

Figure S2. Proportions of enriched biological process (BP) GO terms among highly oxidized transmembrane proteins.

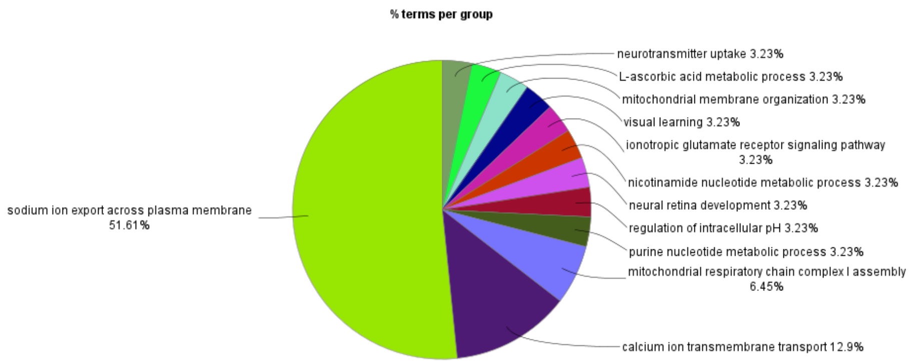

Figure S3. Proportions of enriched cellular component (CC) GO terms among highly oxidized mitochondrial proteins.

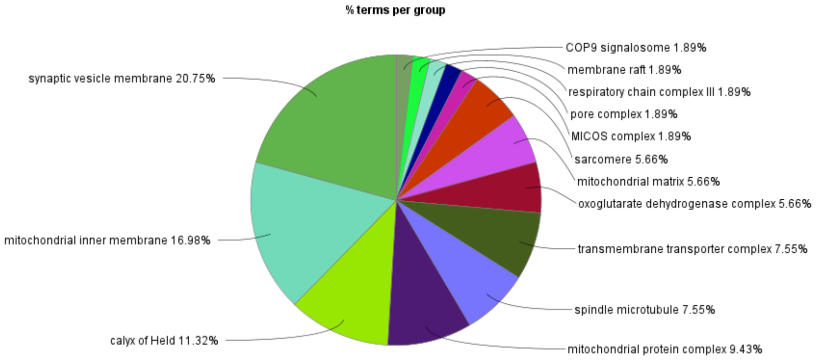

Figure S4. Proportions of enriched cellular component (CC) GO terms among highly oxidized transmembrane proteins.

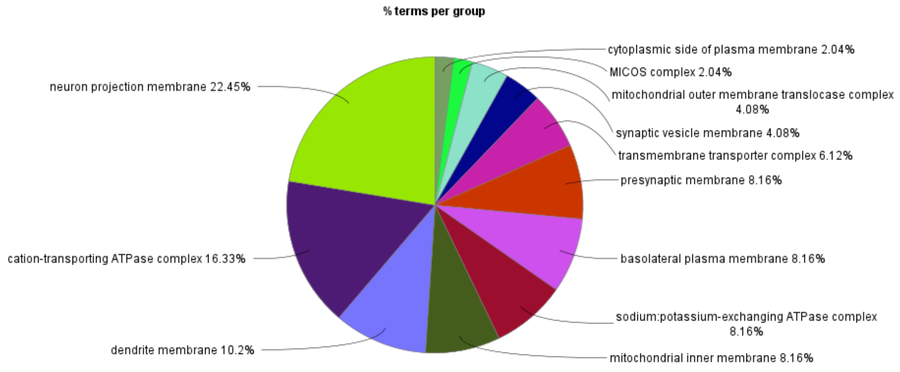

Figure S5. Proportions of enriched molecular function (MF) GO terms among highly oxidized mitochondrial proteins.

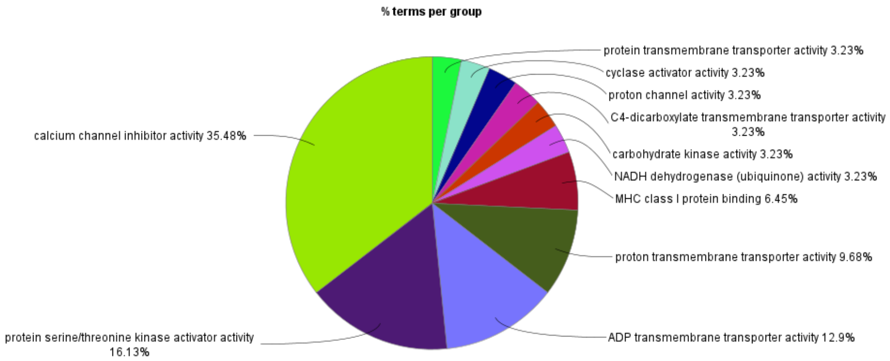

Figure S6. Proportions of enriched molecular function (MF) GO terms among highly oxidized transmembrane proteins.

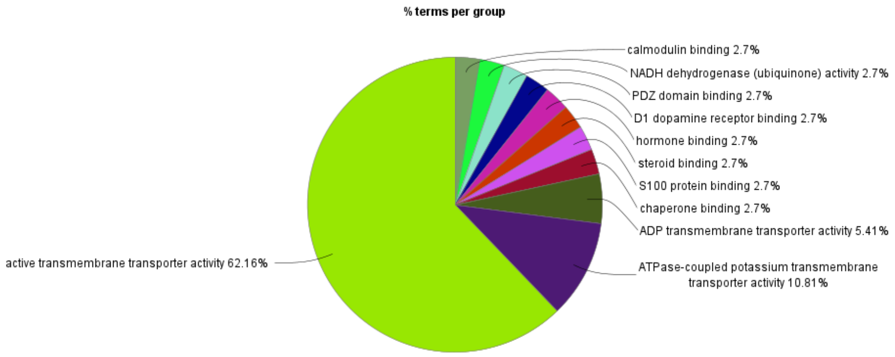

Figure S7. Principal component analysis (PCA) of mono-oxidation profiles in rat cerebrum and heart.

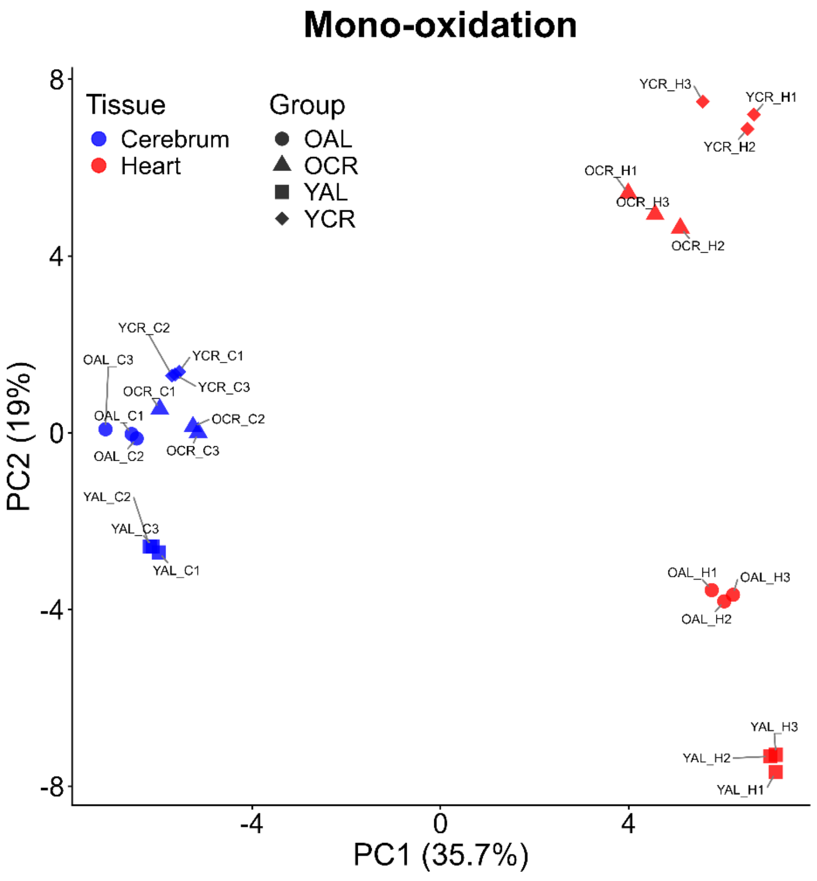

Figure S8. Principal component analysis (PCA) of carbonylation profiles in rat cerebrum and heart.

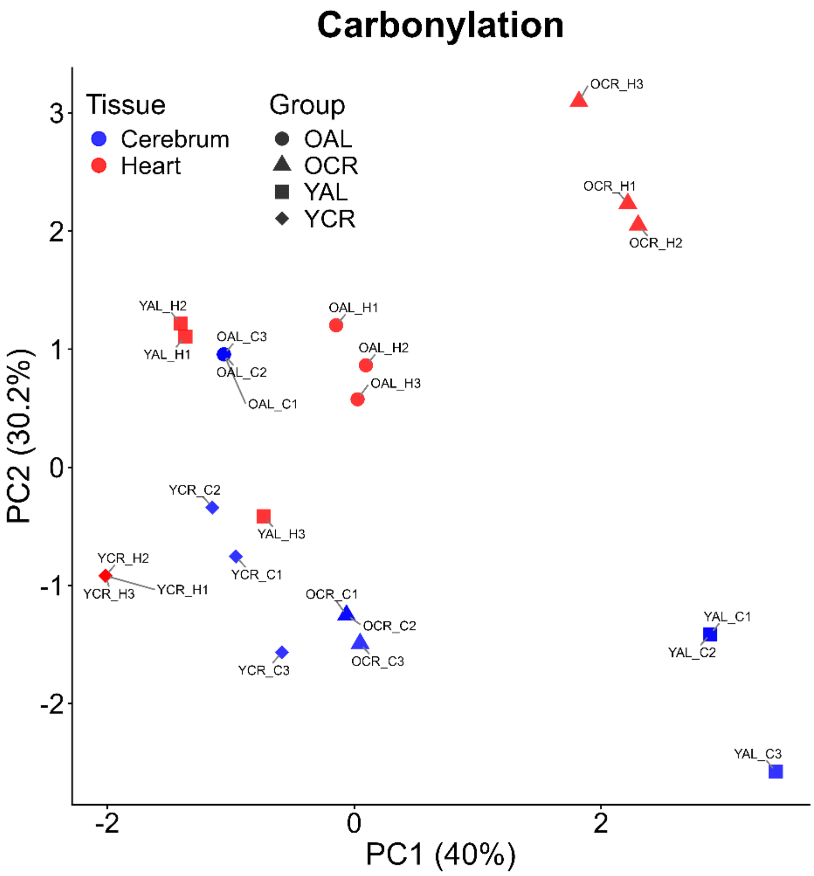

Figure S9. Principal component analysis (PCA) of oxidation(M) modification profiles in rat cerebrum and heart.

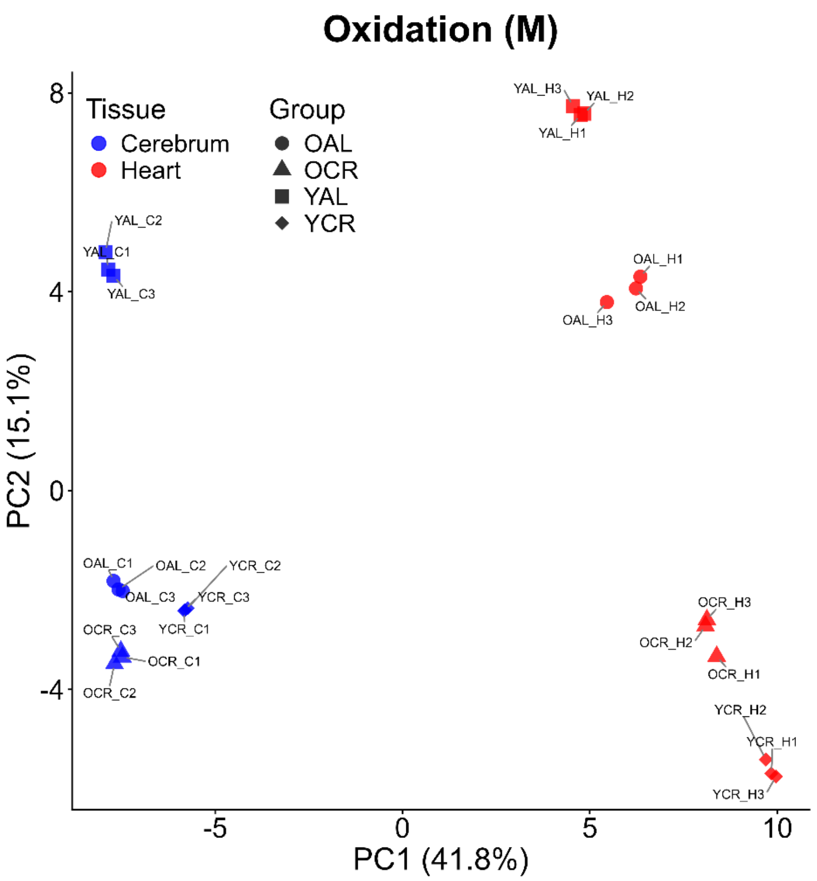

Table S1. Body weights (g) of rats in each experimental group.

Group	n	Individual Body Weights (g)	Mean ± SD (g)
YAL	5	350,351,356,381,360	359.6 ± 12.6
YCR	5	217,196,241,208,211	214.6 ± 16.6
OAL	6	451,314,417,389,416,425	402.0 ± 47.5
OCR	6	211,239,268,260,273,265	252.7 ± 23.6

Note: *YAL = young ad libitum; YCR = young calorie-restricted; OAL = old ad libitum; OCR = old calorie-restricted.*

Table S18. Parameters for database searches and oxidative modification settings in MaxQuant analysis.

Database search	Modification designation	Examination of amino acids	Monoisotopic mass shift (Da)
First	Oxidation	Lys, Asp, His, Leu, Met, Phe, Pro, Trp	+15.99492
	Pyrrolidinone	Pro	-30.01057
	Carbonylation	Arg, Leu, Pro	+13.97927
Second	Kynurenine	Trp	+3.99490
	2-amino-3-ketobutyric acid	Thr	-2.10560
